# Silicon Alleviates the Disease Severity of Sclerotinia Stem Rot in Rapeseed

**DOI:** 10.3389/fpls.2021.721436

**Published:** 2021-09-13

**Authors:** Yuxia Feng, Yuxin Hu, Pengpeng Fang, Xiangjun Zuo, Jinxiong Wang, Jiana Li, Wei Qian, Jiaqin Mei

**Affiliations:** ^1^College of Agronomy and Biotechnology, Southwest University, Chongqing, China; ^2^Academy of Agricultural Sciences, Southwest University, Chongqing, China; ^3^State Cultivation Base of Crop Stress Biology for Southern Mountainous Land of Southwest University, Chongqing, China; ^4^Sichuan Shuangliu Art and Sports Middle School, Chengdu, China; ^5^Hanhong College, Southwest University, Chongqing, China; ^6^Institute of Agricultural Research, Tibet Academy of Agricultural and Animal Husbandry Sciences, Lhasa, China

**Keywords:** *Sclerotinia sclerotiorum*, rapeseed, induced resistance, disease control, silicon

## Abstract

Sclerotinia stem rot, caused by *Sclerotinia sclerotiorum*, is a devastating disease in rapeseed. The objective of this study was to investigate the role and the mechanism of silicon (Si) in alleviating the disease severity of *S. sclerotiorum* in rapeseed. In the absorption assays, the rapeseed that absorbed 10 mM of K_2_SiO_3_ exhibited an 86% decrease in lesion size on infected leaves as compared with controls. In the spray assay, the lesion length on rapeseed stems was reduced by 30.5–32.9% with the use of 100 mM of a foliar Si fertilizer as compared with controls. In the pot assay, the lesion length on rapeseed stems was reduced by 34.9–38.3% when using the Si fertilizer as basal fertilizer. In the field assay, both the disease incidence and disease index of sclerotinia stem rot were significantly reduced with the usage of a solid Si fertilizer, Si foliar fertilizer, and the application of both, without negative affection on the main agronomic traits and seed quality of rapeseed. The transcriptome sequencing, quantitative reverse transcription PCR (qRT-PCR), and biochemical assays between K_2_SO_4_- and K_2_SiO_3_- treated rapeseed leaves revealed that Si promoted the biosynthesis of defense-related substances and enhanced the antioxidation and detoxification abilities of rapeseed after infection. Thus, this study concluded that Si can alleviate the disease severity of *S. sclerotiorum* in rapeseeds, partially due to the induced defense responses.

## Introduction

Rapeseed (*Brassica napus* L., AACC, 2n = 38) is widely cultivated in Canada, Europe, China, and other parts of the world as an important source of both biofuel and edible oil. However, the production of rapeseed suffers from the fungal pathogen *Sclerotinia sclerotiorum* (del Río et al., 2007; Koch et al., 2007), which infects over 600 plant species worldwide (Liang and Rollins, 2018). The disease caused by *S. sclerotiorum*, which is termed as sclerotinia stem rot (SSR), usually results in 10–70% yield losses in rapeseed cultivation (del Río et al., 2007; Koch et al., 2007). Development of resistant lines or cultivars is a cost-effective and sustainable strategy to control to this disease, however, the breeding of resistant rapeseed has been difficult due to the lack of resistant germplasms. Although a few fungicides are available, the low efficiency, environmental contamination, and economic costs cannot be ignored (del Río et al., 2007).

Silicon, which is the second most abundant element on Earth, is a nutrient for plants (Epstein and Bloom, 2005). Numerous studies have reported that Si plays important roles in protecting plants from biotic and abiotic stresses (Fauteux et al., 2005; Datnoff et al., 2007; Debona et al., 2017; Bakhat et al., 2018). Furthermore, it has been strongly suggested that Si plays positive roles in alleviating plant diseases caused by biotrophic or hemibiotrophic pathogens, such as rice blast caused by *Magnaporthe oryzae* (Datnoff et al., 1991; Seebold et al., 2001), powdery mildew in wheat caused by *Blumeria graminis* (Rodgers-Gray and Shaw, 2004), and powdery mildew in pumpkins caused by *Podosphaera xanthii* (Torlon et al., 2016). On the other hand, Si generally exhibits no effect on diseases caused by necrotrophs, such as brown foot rot in wheat caused by *Fusarium culmorum* (Rodgers-Gray and Shaw, 2004), and can even cause heavier infection on plants, such as *Cercospora sojina* in soybean (Nascimento et al., 2014).

The pathogen *S. sclerotiorum* has been considered a typical necrotrophic pathogen for a long time (Purdy, 1979), but biotrophy was recently found from the leading edge of its fungal colonization during the early stages of infection (Kabbage et al., 2015). Until recently, it has been unknown whether Si has any effect on the disease caused by *S. sclerotiorum*. Therefore, this study was carried out to investigate whether Si can alleviate the disease severity of SSR in rapeseed.

## Materials and Methods

### Plant Materials and Fungal Isolate

The rapeseed cultivar “Zhongshuang 11,” which was grown in the experimental field of Southwest University in Chongqing (China), was used in the absorption assay subsequently performed in this study, while the rapeseed cultivar “Westar,” which is susceptible to *S. sclerotiorum*, was used in the spray, pot, and field assays. Additionally, the *S. sclerotiorum* isolate “1980,” which has been sequenced for the genome of *S. sclerotiorum* (Amselem et al., 2011), was used throughout the study. Before inoculation, the sclerotias of “1980” (stored with sterilized wheat at room temperature) were cultured on a potato dextrose agar (PDA) medium for 4 days (at 22°C, without light). Then, the mycelia at the leading edge were transferred to a new PDA medium. Two days later, mycelia-agar discs that were 6 mm in diameter were punched from the growing margin of “1980” and used for inoculation.

### Absorption Assays

In a short-time absorption assay, the third leaves of “Zhongshuang 11” were detached from field-grown plants at the nine-leaf stage and placed on filter papers that were soaked with 10 mM of K_2_SiO_3_ (Guangfu®, Tianjing Guangfu Fine Chemical Research Institute, Tianjin, China), K_2_SO_4_ (Keshi®, Chengdu Kelong Co., Ltd., Sichuan, China; negative control), or ddH_2_O (black control). Accordingly, the petioles of leaves were wrapped with absorbent cotton soaked in 10 mM of K_2_SiO_3_, K_2_SO_4_, or ddH_2_O. These leaves were then investigated for their resistance to the *S. sclerotiorum* isolate “1980” using the detached leaf inoculation method according to Mei et al. (2011). Two mycelia discs were inoculated on each leaf, while all leaves were kept at 22 ± 1°C and ≥85% relative humidity. Three inoculated leaves from each treatment were stained at 24 h after inoculation (hai) using trypan blue according to the study of Frye and Innes (1998) to observe the proliferation of hyphae. The long (*a*) and short (*b*) diameters of each lesion were recorded at 48 hai, and lesion size (*S*) was calculated with the formula: *S* = π*a*b/4 (Mei et al., 2011). The resistance evaluation was carried out five times during December 2019, with five leaves per treatment at each time.

In a long-term absorption assay, the seeds of “Zhongshuang 11” were germinated with moist filter papers. Each of the 30 1-week-old seedlings was moved into a Hoagland's nutrient solution with supplementation of 10 mM of K_2_SiO_3_, K_2_SO_4_, or ddH_2_O, with three biological replications. The plants were kept in a phytotron (16 h of light at 25 ± 1°C/8 h of darkness at 20 ± 1°C), and the liquid solutions were refreshed once a week. After 2 months, the third leaves of the living plants were inoculated with mycelia discs in the laboratory and measured for lesion size at 48 hai. The resistance evaluation was conducted twice, with three replications containing five plants per replication per treatment.

### Spray Assay

The spray assay was conducted on both the leaves and stems of the rapeseeds. The detached leaves of “Westar” (field-grown plants, nine-leaf stage) were sprayed with 100 mM of K_2_SiO_3_ (5 ml per five leaves) and then inoculated with the mycelia discs of *S. sclerotiorum* in the lab according to the study conducted by Mei et al. (2011). The sprays of the ddH_2_O and K_2_SO_4_ solutions on detached leaves were set as the black control and the negative control, respectively. Each leaf was inoculated with one mycelia disc, and all leaves were kept at 22 ± 1°C and ≥85% relative humidity. Lesion size was recorded at 48 hai. The experiment was conducted seven times, with 10–20 leaves per treatment at each time. The stems of “Westar” were detached 2 weeks before harvest and sprayed with ddH_2_O, 100-mM K_2_SiO_3_, or K_2_SO_4_ solutions (5 ml per three stems) and then inoculated with mycelia discs in the laboratory according to the study conducted by Mei et al. (2012). Each stem was inoculated with two mycelia discs. The inoculated stems were kept at 22 ± 1°C and ≥85% relative humidity. The length (*l*) of each lesion was measured at 48 hai. The experiment was conducted three times, with nine stems in each treatment.

### Pot Assay

In the pot assay, each of the 12 4-week-old seedlings of “Westar” was transplanted from the nursery into a 15 cm-diameter plastic pot, which was filled with Pindstrup substrate No. 5 (Pindstrup, Ryomgård, Denmark), 1.15 g/pot of a potassium sulfate compound fertilizer (K_2_O ≥ 52%, Luo-Bu-Po®, SDIC Xinjiang Luobupo Potash Co., Ltd., Xinjiang, China) + 1.2 g/pot of a solid Si fertilizer (SiO_2_ ≥ 20%, Gaofei Agriculture Co., Ltd., Wuhan, China), 2.35 g/pot of a potassium sulfate compound fertilizer (as the negative control), or 1.15 g/pot of a potassium sulfate fertilizer compound fertilizer (as the black control) as a base fertilizer. All the 12 pots of each treatment were placed on a 60 × 40 × 10 cm plastic plate and kept in a greenhouse at 25°C during the day and 20°C during the night. All the plants were complemented with 1.15 g/pot of a potassium sulfate compound fertilizer before flowering. Four biological replications were conducted. The third leaves of the living plants at nine-leaf stage were inoculated in the laboratory and measured for lesion size at 48 hai, while the stem resistance was tested 2 weeks before harvest using detached stem inoculation. The inoculated plants or stems were kept at 22 ± 1°C and ≥85% relative humidity. The lesion size on the leaves and the lesion length on the stems were both measured at 48 hai. The resistance evaluation on the leaves was conducted seven times, with seven leaves tested in each treatment each time. The stem inoculation was carried out six times, with four stems tested in each treatment each time.

### Field Assay

The field experiment was conducted in two crop seasons, during 2020 and 2021, in the experimental field of Southwest University. Each of 80 plants of “Westar” was transplanted from the nursery into a 3 × 2 m plot in 2020, with 30 cm between rows and 25 cm within rows, while each of 40 plants was planted in a 1.5 × 2 m plot in 2021. Two factors were designed, i.e., the use of a solid fertilizer as a base fertilizer (applied when transplanting) and the use of liquid fertilizer as a foliar fertilizer during bloom (sprayed three times, with 1-day intervals). In detail, four treatments were designed (Table 1), including (1) 33 g/m^2^ of a solid potassium sulfate fertilizer + 200 ml/m^2^ of a 100-mM liquid potassium sulfate fertilizer (noted as S_solid_ + S_liquid_), (2) 33 g/m^2^ of a solid potassium sulfate fertilizer + 200 ml/m^2^ of a 100-mM liquid Si fertilizer (chelated Si ≥ 99%, Meixinlvgaole®, Donglixin Co., Ltd., Henan, China) (noted as S_solid_ + Si_liquid_), (3) 15 g/m^2^ of a solid potassium sulfate fertilizer amended with 18 g/m^2^ of a solid Si fertilizer + 200 ml/m^2^ of a 100-mM liquid potassium sulfate fertilizer (noted as Si_solid_ + S_liquid_), and (4) 15 g/m^2^ of a solid potassium sulfate fertilizer amended with 18 g/m^2^ of a solid Si fertilizer + 200 ml/m^2^ of a 100-mM liquid Si fertilizer (noted as Si_solid_ + Si_liquid_). A two-factor randomized design was applied in the field assay. Five and four replications were conducted in the first and the second crop season, respectively, with one plot per treatment in each replication.

**Table 1:** Descriptions of the treatments and the phenotypic data of the rapeseed in the field experiment.

**Treatment**	**Solid fertilizer (g/m** ^**2**^ **)**		**Liquid fertilizer** **(mL/m** ^**2**^ **)**		**Lesion length (cm)**	**Disease incidence(%)**	**Disease index**	**RCE^#^ (%)**	**Plant height (cm)**	**Number of branches**	**Seeds per pod**	**1000-seed weight (g)**
	**K_**2**_SO_**4**_**	**Si**	**K_**2**_SO_**4**_**	**Si**								
S_solid_ + S_liquid_	33	–	200	–	7.3 ± 1.04A^*^	58.0 ± 11.48A	45.8 ± 5.71A	–	184.9 ± 9.70A	7.3 ± 1.00A	19.3 ± 4.07A	3.6 ± 0.11A
S_solid_ + Si_liquid_	33	–	–	200	6.9 ± 1.14A	38.1 ± 6.10B	31.2 ± 4.33B	31.9	183.2 ± 10.33A	7.6 ± 1.51A	19.0 ± 3.15A	3.5 ± 0.14A
Si_solid_ + S_liquid_	15	18	200	–	6.4 ± 1.28B	36.1 ± 7.37B	28.1 ± 4.64BC	38.6	184.1 ± 9.40A	7.6 ± 1.03A	19.7 ± 3.97A	3.5 ± 0.18A
Si_solid_ + Si_liquid_	15	18	–	200	5.8 ± 1.73C	32.9 ± 7.93B	23.2 ± 4.92C	49.3	183.6 ± 10.66A	7.6 ± 0.92A	19.3 ± 4.01A	3.6 ± 0.10A
MSE^#^	–	–	–	–	0.10	2.27	1.72	–	0.77	0.66	0.09	0.01

# *RCE, relative control efficiency comparing to treatment S_solid_ + S_liquid_; MSE, mean square error*.

* *The data (M ± SD) with different capital letters in the same column indicate significant differences at the p = 0.05 level by the Duncan range test. Disease incidence and disease index data came from 2020 and 2021 field assays. Lesion length data came from the 2020 field assay. The plant height, number of branches, seeds per pod, and 1,000-seed weight were only covered in 2021*.

The disease incidence (DIN) and disease index (DI) of SSR under natural infections were investigated in both 2020 and 2021. According to the national agricultural standard for the identification of resistance to *S. sclerotiorum* in rapeseed (Oil Crops Research Institute, 2016), the plants were rated for disease grades (*si*) at 1 week before harvest on a 0–4 scale, where *s0* meant no visible symptoms on the whole plant; *s1* represented visible symptoms on ≤ one-third of the branches or a ≤ 3-cm lesion on the main stem with ≤ one-fourth of the siliques being infected; *s2* meant visible symptoms on approximately one- to two-thirds of the branches, or visible symptoms on ≤ one-third of the branches but with a >3 cm lesion on the middle and upper parts of the main stem, with approximately one-fourth to half of the siliques being infected; *s3* meant visible symptoms on ≥ two-thirds of the branches, or visible symptoms on < two-thirds of the branches but with a >3 cm lesion on the middle and basal parts of the main stem, with approximately half to three-fourths of the siliques being infected; and *s4* meant symptoms on nearly all the branches, or multiple lesions on the main stem, with > three-fourths of the siliques being infected. The disease index was calculated according to the formula DI = $\frac{\sum \left(si\times ni\right)}{N\times 4}\times 100$, where *ni* is the number of plants in corresponding *si* and *N* is the total number of plants. On the other hand, DIN was recorded as the percentage of diseased plants in the total number of plants. The relative control efficiency (RCE) when comparing with treatment S_solid_ + S_liquid_ was calculated according to the formula: RCE (%) = (DI_Ssolid+Sliquid_ – DI_treatment_)/DI_Ssolid+Sliquid_ × 100. A detached stem inoculation was carried out in 2020 at 2 weeks before harvest. Ten stems per treatment in each replication were tested. The length of each lesion was measured at 72 hai. The plant height, the number of branches, the seed set of open-pollinated plants, and the 1,000-seed weight were investigated in 2021 in 10–30 symptom-free plants before harvest. Seed quality was determined by near-infrared reflectance spectroscopy (NIRS) from five open-pollinated plants in each treatment, with three technical replicates.

### Physiological and Biochemical Assays

The silicon content (*C*) in the leaf was measured using the Si molybdenum blue colorimetry method (Yang et al., 2015). The lignin concentration (*L*) of the leaf was measured according to Robinson and Mansfield (2009). The glutathione peroxidase activity (*GPx*) was analyzed using the Total Glutathione Peroxidase Assay Kit (Beyotime, Jiangsu, China) following the instructions of the manufacturer. Total antioxidant capacity (*A*) was tested using a 2,2-azino-bis-3-ethylbenzothiazoline-6-sulfonic acid (ABTS) method with the Total Antioxidant Capacity Assay Kit (Beyotime, China) (Mei et al., 2016). Finally, all these assays were conducted with three biological replications, with five samples tested in each replication.

### Transcriptome Sequencing

Transcriptome sequencing (RNA-Seq) was carried out among four rapeseed leaf samples, i.e., the leaves that absorbed 10 mM of K_2_SO_4_ (L-S) and 10 mM of K_2_SiO_3_ (L-Si) for 24 h and the corresponding *S. sclerotiorum*-inoculated leaves sampled at 24 hai (L-S-s and L-Si-s, respectively). The plant materials and the techniques of absorption and inoculation were consistent with the short-time absorption assay. Total RNA was extracted using the Eastep® Supper kit (Promega, Shanghai, China). The six libraries were sequenced on the Illumina Hiseq 2000^TM^ platform in Biomarker Technologies (Beijing, China). The row data were deposited in the Sequence Read Archive (SRA) database (BioProject ID PRJNA749126). Clean reads were aligned to a rapeseed reference genome (ftp://ftp.ncbi.nlm.nih.gov/genomes/all/GCF/000/686/985/GCF_000686985.2_Bra_napus_v2.0). The expressions of all the unigenes were estimated using reads per kilobyte per million mapped reads (RPKM) (Mortazavi et al., 2008). Furthermore, differentially expressed genes (DEGs) were identified according to the study of Dang et al. (2013). The threshold determining the significance of these DEGs among multiple tests was set at a false discovery rate (*FDR*) of ≤ 0.001 and |log_2_(folds change)| of ≥1. Functional enrichment analyses such as the Kyoto Encyclopedia of Genes and Genomes (KEGG) and Gene Ontology (GO) analyses were performed using the ultra-geometric test (Du et al., 2010). An *FDR*-value of ≤ 0.05 was set as the threshold to determine the significant enrichment of GO terms and KEGG pathways. A heatmap of interested genes was constructed by using the software package “Heatmap” on the BMKCloud platform (www.biocloud.net).

### Quantitative Reverse Transcription PCR

Reverse transcription was conducted using the FastQuant RT SuperMix (TIANGEN, Beijing, China). The qPCR amplification was performed using 2 × SYBR Green qPCR Master Mix (US Everbright® Inc., Suzhou, China) on a CFX96 Touch Deep Well™ Real-Time PCR Detection System (Bio-Rad, Hercules, CA, USA) with three biological replications. Differential gene expression was calculated using the 2^−ΔΔCt^ method. The *BnActin7* (NM_001316010.1) was used as an internal reference control. The primer sequences for all the genes are available in Supplementary Table 1.

### Statistical Analysis

Analysis of variance was performed separately in various trials on phenotypic data using Proc GLM in SAS software (SAS Institute, 2000). Duncan's multiple comparisons were applied to test the difference among treatments.

## Results

### Silicon Increased Resistance to *S. sclerotiorum* in Rapeseed in the Laboratory

**Figure 1:**
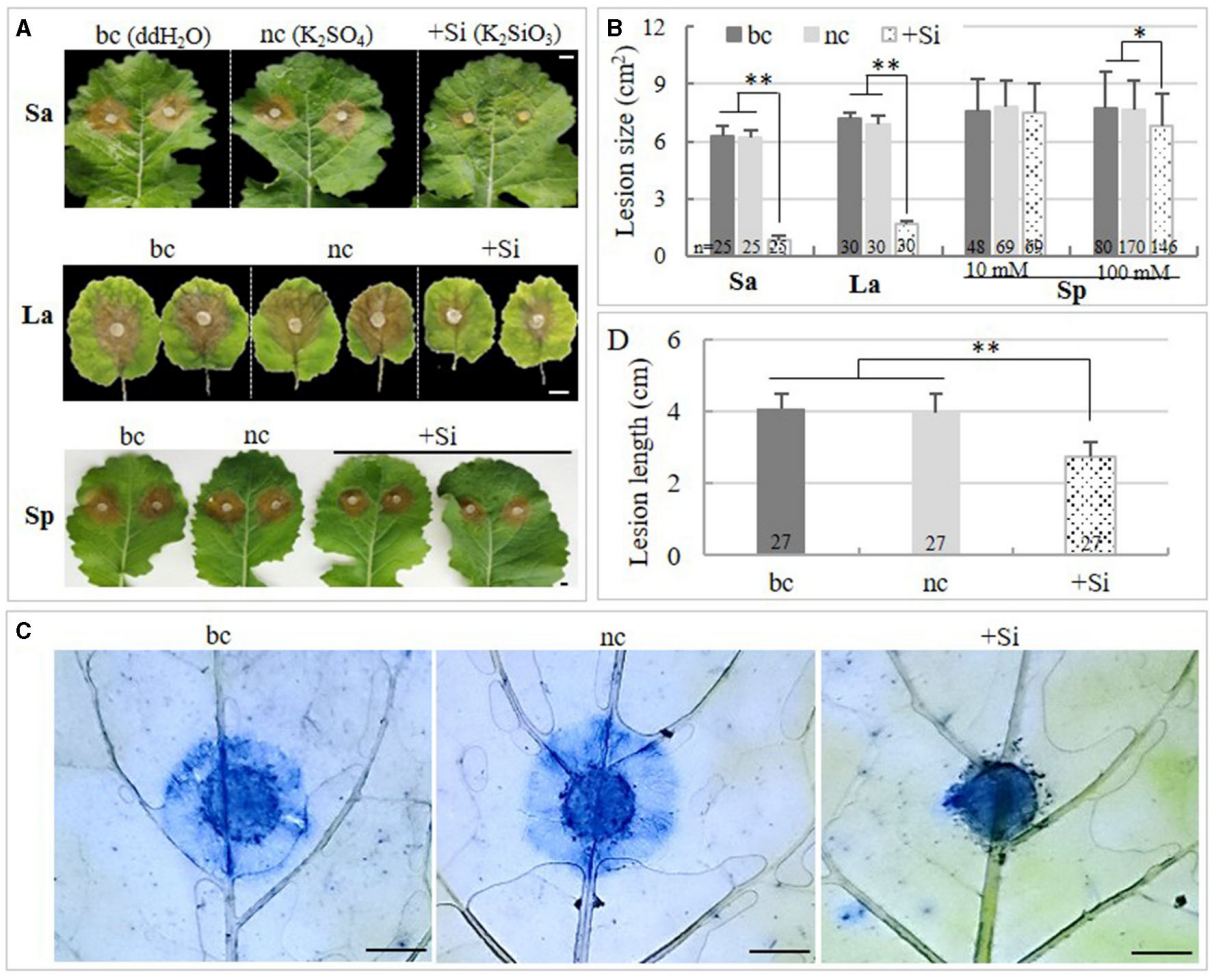
Effects of K_2_SiO_3_ on plant resistance to *Sclerotinia sclerotiorum* in absorption and spray assays. **(A)** Disease symptoms of inoculated rapeseed leaves under the K_2_SiO_3_ treatment (+Si), K_2_SO_4_ treatment (the negative control, nc), and ddH_2_O treatment (the black control, bc). Sa: short-time absorption assay; La: long-term absorption assay; Sp: spray assay. Bars = 1 cm. **(B)** Comparison of lesion size on the inoculated leaves of rapeseed plants among treatments. **(C)** Comparison on the proliferation of *S. sclerotiorum* on rapeseed leaves at 24 hai among treatments. The trypan blue staining was applied, and mycelia and necrosis were stained in blue. Bars = 5 mm. **(D)** Comparison of lesion length on the stems of the rapeseeds among treatments in the spray assay. The number on the bottom of each bar in **(B)** and **(D)** represents the number of samples tested. ^*^ and ^**^ indicate significant differences at the *p* < 0.05 and *p* < 0.001 levels, respectively.

Absorption experiments, which could block the contact between the solution and the pathogen, were conducted in the laboratory to investigate the effect of Si on rapeseed. In the short-time absorption experiment, the lesion size was reduced by 85.5 and 85.7% on leaves that absorbed 10 mM of K_2_SiO_3_ as compared with the black and the negative control, respectively (*S*_*ddH*2*O*_= 6.3 ± 0.55 cm^2^, *S*_*K*2*SO*4_ = 6.2 ± 0.36 cm^2^, *S*_*K*2*SiO*3_ = 0.9 ± 0.22 cm^2^; *p* < 0.001) (Figures 1A,B). The staining assay revealed that the proliferation of the pathogen was significantly suppressed in leaves that absorbed K_2_SiO_3_, as compared with those treated with K_2_SO_4_ and ddH_2_O (Figure 1C). Consistently, in the long-term (2 months) absorption assay, the lesion size on plants supplemented with K_2_SiO_3_ was reduced by 76.4 and 75.4% as compared with the black and the negative control, respectively (*S*_*K*2*SiO*3_= 1.7 ± 0.13 cm^2^, *S*_*ddH*2*O*_ = 7.2 ± 0.28 cm^2^, *S*_*K*2*SO*4_ = 6.9 ± 0.42 cm^2^) (Figures 1A,B). The plants supplemented with K_2_SiO_3_ also showed higher Si content in the leaves (*C*_*si*_ = 38.5 ± 2.53 mg/kg·fresh weight [FW]) as compared with plants cultured with ddH_2_O (*C*_*si*_ = 12 ± 1.77 mg/kg·FW) and K_2_SO_4_, (*C*_*si*_ = 10.3 ± 3.92 mg/kg·FW) (*p* < 0.001).

**Figure 2:**
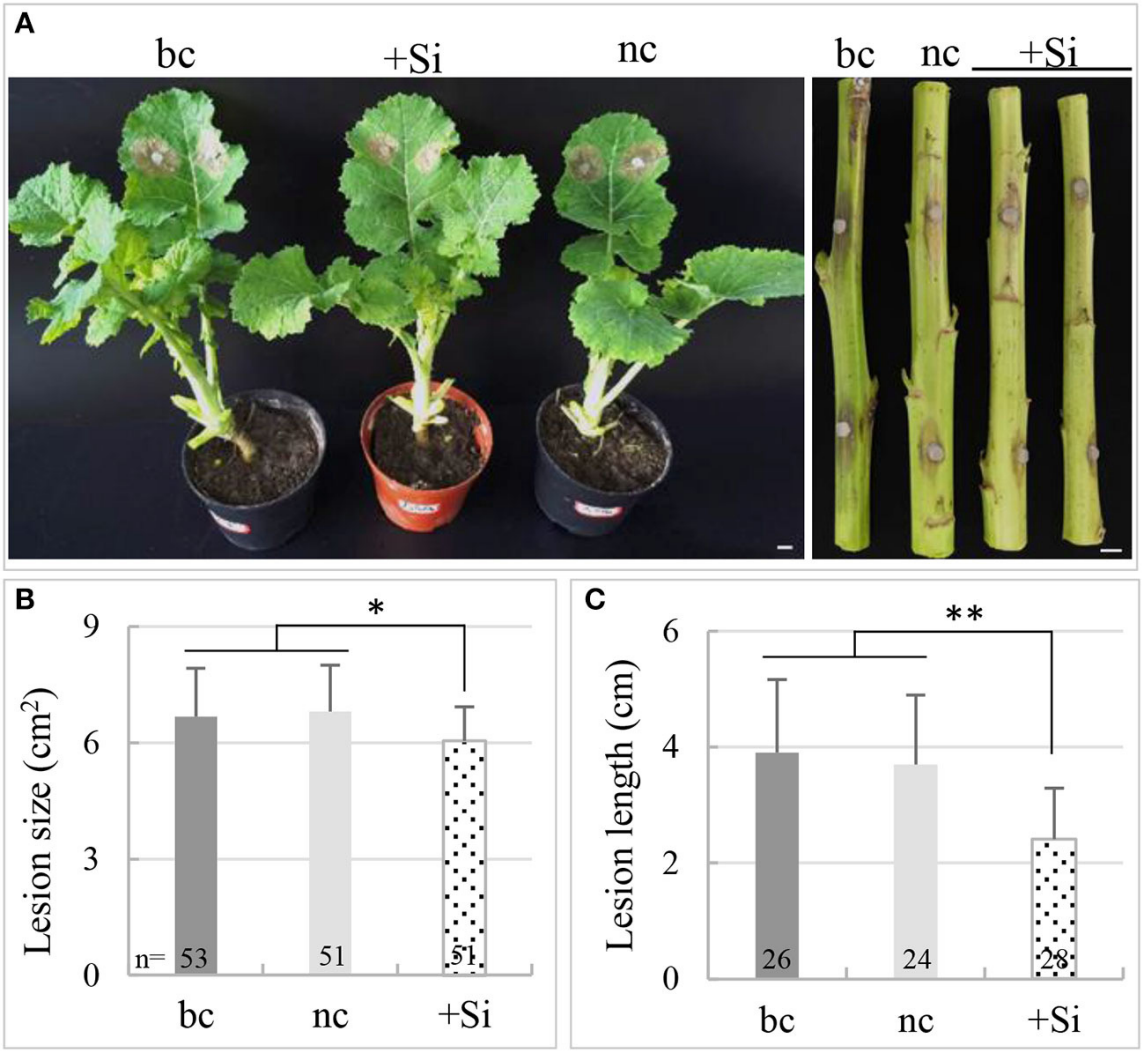
Effects of the silicon (Si) fertilizer on plant resistance to *S. sclerotiorum* in a pot assay. **(A)** Disease symptoms of inoculated rapeseed under +Si treatment (Si fertilizer), potassium sulfate fertilizer treatment (the negative control, nc), and the black control (bc). Bars = 1 cm. **(B)** Comparison of lesion size on the inoculated leaves of rapeseed among treatments. **(C)** Comparison of lesion length on the inoculated stems of rapeseed among treatments. The number on the bottom of each bar of B and C represent the number of samples tested. * and ** indicate significant differences at the *p* < 0.05 and *p* < 0.001 levels, respectively.

Since both the short-time and the long-term absorption of Si could increase the resistance of rapeseed to *S. sclerotiorum*, a spray assay and a pot assay were carried out to investigate the effect of a liquid Si (transient spray) and solid Si basal fertilizer (long-term absorption) on enhancing resistance to *S. sclerotiorum* in rapeseed, respectively. It was found that, as compared with the negative control, the lesion size on the leaves and the lesion length on the stems were reduced by 10 and 30.5%, respectively, by spraying 100 mM of K_2_SiO_3_ solution (*p*_*leaf*_ < 0.05, *p*_*stem*_ < 0.0001) (Figures 1A,B,D). The same was reduced by 11 and 34.9% with the use of a solid Si fertilizer in the pot assay (*p*_*leaf*_ < 0.05, *p*_*stem*_ < 0.0001) (Figure 2). This suggested that both the transient spray of liquid Si and using the solid Si basal fertilizer had a protective role in the resistance of rapeseed against *S. sclerotiorum*, particularly in the stems.

### Silicon Fertilizer Alleviated the Disease Severity of SSR in Rapeseed in the Field

In the field experiment, the solid and liquid Si fertilizers were tested separately or collaboratively for their effects on alleviating the disease severity of SSR. The stem resistance was evaluated in the first crop season, revealing significant decreases in lesion length using the treatment Si_solid_ + Si_liquid_ (*l* = 5.8 ± 1.73 cm) and the treatment Si_solid_ + S_liquid_ (*l* = 6.4 ± 1.28 cm) compared with control (treatment S_solid_ + S_liquid_, *l* = 7.3 ± 1.04 cm) (*p* < 0.0001), while treatment S_solid_ + Si_liquid_ (*l* = 6.9 ± 1.14 cm) showed no difference with control (*p* > 0.05) (Table 1). The DIN and DI were recorded in both 2020 and 2021, without significant differences across years (*p*_*DIN*_ = 0.8588, *p*_*DI*_ = 0.634) and among replications (*p*_*DIN*_= 0.5148, *p*_*DI*_ = 0.974). Comparing to treatment S_solid_ + S_liquid_ (DIN = 58 ± 11.48%, DI = 45.8 ± 5.71), the DIN and DI were significantly reduced with the application of the solid Si fertilizer (treatment Si_solid_ + S_liquid_, DIN = 36.1 ± 7.37%, DI = 28.1 ± 4.64), the spray of the liquid Si fertilizer (treatment S_solid_ + Si_liquid_, DIN = 38.1 ± 6.1%, DI = 31.2 ± 4.33), and, in particular, the application of both (treatment Si_solid_ + Si_liquid_, DIN = 32.9 ± 7.93%, DI = 23.2 ± 4.92) (*p*_*DIN*_ < 0.0001, *p*_*DI*_ < 0.0001) (Table 1). The relative control efficiency of the solid Si fertilizer, liquid Si fertilizer, and solid Si fertilizer + liquid Si fertilizer was 38.6, 31.9, and 49.3%, respectively (Supplementary Figure 1). In addition, the use of the Si fertilizer was found to have no affection on rapeseed in terms of plant height (*p* = 0.858), the number of branches (*p* = 0.430), the seed set of open-pollinated plants (*p* = 0.879), the 1,000-seed weight (*p* = 0.079), and seed quality (Table 1).

### Silicon Promoted Defense Response in Rapeseed Under *S. sclerotiorum* Infection

**Figure 3:**
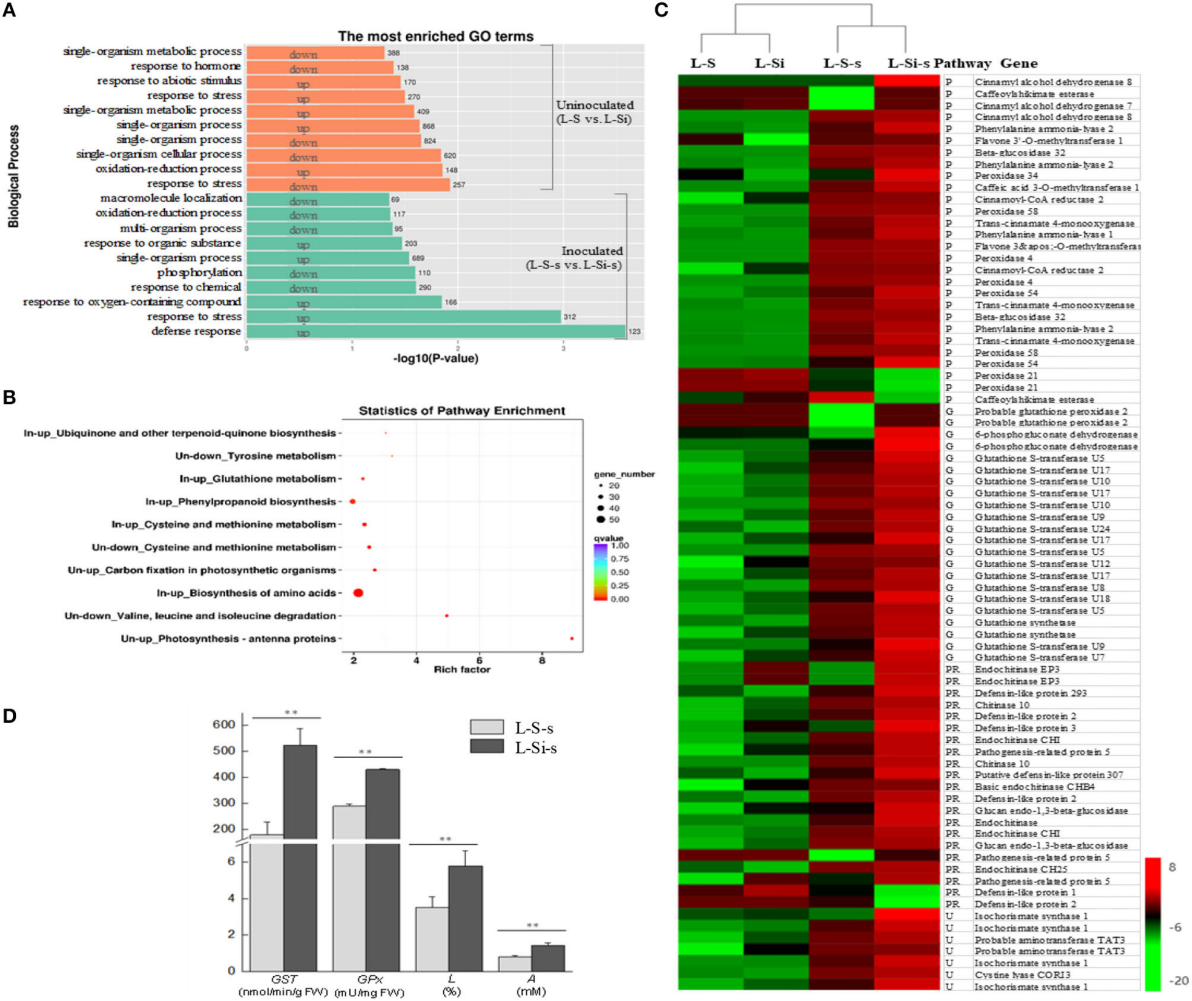
Transcriptional differences of rapeseed leaves between K_2_SiO_3_ and K_2_SO_4_ treatments (K_2_SO_4_ vs. K_2_SiO_3_). **(A)** The most enriched Gene Ontology (GO) terms from the up- and downregulated differentially expressed genes (DEGs) in K_2_SiO_3_-treated leaves before inoculation (uninoculated) and at 24 hai (inoculated). The number on the right side of each bar indicates the number of DEGs. **(B)** Kyoto Encyclopedia of Genes and Genomes (KEGG) enrichments from the up- and downregulated DEGs in K_2_SiO_3_-treated leaves before inoculation (uninoculated, abbreviated as “Un” in the figure) and at 24 hai (inoculated, abbreviated as “In” in the figure). **(C)** Heatmap of interested genes in uninoculated and inoculated rapeseed leaves under K_2_SiO_3_ and K_2_SO_4_ treatments according to the reads per kilobyte per million mapped reads (RPKM) values. P, phenylpropanoid biosynthesis; G, glutathione metabolism; U, ubiquinone and other kinds of terpenoid-quinone biosynthesis; PR, pathogenesis-related genes. **(D)** The glutathione peroxidase activity (*GPx*), lignin content (*L*), and total antioxidant capacity **(A)** of the pathogen-inoculated rapeseed leaves that absorbed K_2_SiO_3_ (L-Si-s) and K_2_SO_4_ (L-S-s). ** *p* < 0.05.

In order to understand the general mechanism of Si when enhancing resistance to *S. sclerotiorum* in rapeseed, RNA-Seq was conducted in rapeseed leaves that absorbed 10 mM of K_2_SiO_3_ and K_2_SO_4_ for 24 h (sample L-Si and L-S, uninoculated) and in pathogen-inoculated leaves at 24 hai (sample L-Si-s and L-S-s). Over 20 million clean reads were yielded for each sample, and over 91% were aligned to the reference genome of *B. napus* (Supplementary Table 2). Without pathogen inoculation, 4,083 DEGs were detected between the leaves treated by K_2_SiO_3_ and K_2_SO_4_, including 2,040 downregulated (Si-repressed) genes and 2,043 upregulated (Si-induced) genes. These DEGs were significantly enriched in a few GO and KEGG items related to amino metabolism, carbon fixation, and photosynthesis (such as “valine, leucine, and isoleucine degradation,” “cysteine and methionine metabolism,” “tyrosine metabolism,” “photosynthesis,” and “carbon fixation in photosynthetic organisms”) (Figures 3A,B). After inoculation, 1,639 genes were downregulated in K_2_SiO_3_-treated leaves as compared with the K_2_SO_4_-treated leaves, i.e., Si-repressed genes responding to *S. sclerotiorum*, without significant GO and KEGG enrichments that were directly involved in defense response against pathogens. In contrast, 2,344 upregulated DEGs in K_2_SiO_3_-treated leaves, i.e., Si-induced genes responding to the pathogen, were found to significantly enrich several defense-related GO terms and KEGG pathways, such as “defense response,” “phenylpropanoid biosynthesis,” “glutathione metabolism,” and “ubiquinone and other kinds of terpenoid-quinone biosynthesis” (Figures 3A,B).

Genes involved in the defense-associated GO terms and KEGG pathways were further analyzed among the four samples. In detail, 25 in 28 DEGs involved in “phenylpropanoid biosynthesis” were upregulated in the sample L-Si-s (K_2_SiO_3_-treated leaf at 24 hai) as compared with the sample L-S-s (K_2_SO_4_-treated leaf at 24 hai), including 11 genes involved in lignin biosynthesis, such as cinnamyl alcohol dehydrogenase and flavone 3'-O-methyltransferase (Figure 3C). All 22 DEGs involved in “glutathione metabolism” were upregulated in L-Si-s, containing 16 glutathione S-transferases (GSTs), 2 glutathione synthetases, and 2 glutathione peroxidases (GPxs) (Figure 3C). Similarly, all seven DEGs involved in “ubiquinone and other kinds of terpenoid-quinone biosynthesis” were upregulated in L-Si-s (Figure 3C). In addition, 19 of the 21 differentially expressed pathogenesis-related (PR) genes were upregulated in L-Si-s (Figure 3C). Most of the genes mentioned above exhibited low expression levels in the two uninoculated samples. The qRT-PCR analysis for 12 DEGs described above showed agreement with the RNA-seq data (Supplementary Figure 2).

The transcriptional differences were further confirmed by biochemical assays, in which the sample L-Si-s showed significantly higher values than the sample L-S-s (*p* < 0.05) on lignin content (*L*_*K*2*SiO*3_ = 5.8 ± 0.83%, *L*_*K*2*SO*4_ = 3.5 ± 0.61%), GST activity (*GST*_*K*2*SiO*3_ = 523.7 ± 64.4 nmol/min/g·FW, *GST*_*K*2*SO*4_ = 178 ± 48.5 nmol/min/g·FW), GPx activity (*GPx*_*K*2*SiO*3_ = 429.7 ± 4.13 mU/mg·FW, *GPx*_*K*2*SO*4_ = 288.2 ± 8.88 mU/mg·FW), and total antioxidant capacity (*A*_*K*2*SiO*3_ = 1.4 ± 0.8 mM, *A*_*K*2*SO*4_ = 0.8 ± 0.05 mM) (Figure 3D).

## Discussion

Silicon is not only a nutrient for plants but also plays positive roles in protecting plants from biotic and abiotic stresses (Fauteux et al., 2005; Datnoff et al., 2007; Debona et al., 2017; Bakhat et al., 2018). However, no in-depth research study has been conducted so far on how Si supplementation can improve the resistance of host plants to *S. sclerotiorum*. Experience has repeatedly shown that Si has no effect on this pathogen (Coskun et al., 2019), which had been thus far considered as a typical necrotrophic pathogen (Purdy, 1979) before the reveal of its biotrophic phase (Kabbage et al., 2015). This study provided evidence on the positive role of Si in alleviating the disease severity caused by *S. sclerotiorum* in rapeseed. To our knowledge, this is the first report linking the beneficial role of Si to host defense against *S. sclerotiorum*.

The theory concerning the action mode of Si in plant prophylaxis involved physical barriers (Kim et al., 2002; Cai et al., 2008) and biochemical defense reactions under pathogen stresses (Cai et al., 2008; Tatagiba et al., 2014; Song et al., 2016; Wang et al., 2017). In this study, the rapeseed leaves that absorbed the K_2_SiO_3_ solution, which blocked the direct contact between K_2_SiO_3_ and the pathogen, had a large decrease (around 86%) in lesion size as compared with the leaves that received the K_2_SO_4_ treatment. This clearly suggests that Si contributed to the defense system of the rapeseeds. The transcriptome analysis and biochemical assays revealed that K_2_SiO_3_ significantly promoted the expression of a large set of “defense response” genes in response to *S. sclerotiorum*, including genes encoding PR proteins and genes involved in lignin biosynthesis, the glutathione metabolism (such as GSTs and GPxs), and the biosynthesis of ubiquinone and other terpenoid-quinone. Pathogenesis-related proteins are generally induced by various types of pathogens, most of which exhibit antifungal activity (Sinha et al., 2014). Lignin, which deposits in the secondary cell wall, has been suggested to be a physical barrier against pathogens (Sattler and Funnell-Harris, 2013; Cesarino, 2019). Glutathione S-transferases display significant roles in the detoxification of toxic substances and the attenuation of oxidative stress in the plant during pathogen infections (Gullner et al., 2018). Glutathione peroxidases catalyze the reduction in H_2_O_2_, organic hydroperoxides, and lipid peroxides (Ursini et al., 1995). Ubiquinone (Coenzyme Q) plays a role in antioxidant defense response (Bentinger et al., 2010). Thus, the data of this study suggest that Si promoted host defense responses to *S. sclerotiorum*, such as the biosynthesis of defense-related substances and the enhancement of host antioxidation and detoxification abilities, which consequently contributed to the resistance of rapeseed to this pathogen. Similarly, Si also augmented antioxidant components and the detoxification system to enhance the resistance in Brassica species against abiotic stresses, such as heavy metals (cadmium and arsenic) in *B. napus, B. chinensis*, and *B. juncea* and salinity and drought stresses in *B. juncea* (Alamri et al., 2020). It is interesting that, without pathogen infection, K_2_SiO_3_ did not significantly alter the expression of the defense-related genes mentioned above. It was reported that, like secondary messengers, the effects of Si on secondary metabolisms are significant only after pathogen elicitation; thus, Si may act as a modulator influencing the timing and extent of plant defense responses in a manner reminiscent of secondary messengers (Fawe et al., 2001; Fauteux et al., 2005). Besides the aforementioned mechanism, it is worth mentioning that Si possibly has a direct effect on the pathogen since the growth of the hyphae was entirely inhibited by K_2_SiO_3_ with a concentration above 12.5 mM (added into the culture medium of *S. sclerotiorum*). Further study will be carried out to reveal the fungistatic or fungicidal effect of Si on *S. sclerotiorum*.

In this study, Si was found to alleviate disease severity in rapeseed no matter Si was applied in a transient (spray of liquid Si) or long-term way (conscious supplication of Si, such as the absorption assays, the pot assay, and the field assay). However, the long-term supplementation of Si seemed more effective than the transient application in terms of protecting rapeseed from *S. sclerotiorum*. The first clue is that the spray of 100 mM of the K_2_SiO_3_ solution exhibited a similar effect as the use of 10 mM of K_2_SiO_3_ in the liquid culture, while a spray of 10 mM of K_2_SiO_3_ was ineffective in decreasing symptoms (Figure 1B). The second proof is that the relative control efficiency by spraying liquid Si fertilizer on the field plants (treatment S_solid_ + Si_liquid_, RCE = 31.9%) was less than that when using the Si basal fertilizer (treatment Si_solid_ + S_liquid_, RCE = 38.6%), even though the 100 mM of liquid fertilizer was sprayed three times. One possible reason is that a proportion of Si may have been lost after spraying due to evaporation. More possibly, the surface of the shoots (the up-ground part) could have had low Si-uptake efficiency. In rice, which is a strong Si-accumulator, the uptake of Si takes place in the roots, where a specific low Si 1 (LSi1) channel (Ma et al., 2006) is involved and then translocated to the shoots by other Si transporters (Ma et al., 2007).

Silicon is the second most abundant element on Earth. It is inexpensive, environmentally friendly, and readily available. The latest definition of essential nutrients has defined Si as essential for plants (Epstein and Bloom, 2005). Although rapeseed is classified as a non-Si accumulator species, putative Si-efflux transporters were recently identified in this species (Sonah et al., 2017), and rapeseed was found to be able to accumulate Si under a high concentration of Si in the soil (Diana et al., 2021) and 1-week supplication of Si *via* liquid culture (Haddad et al., 2018). In addition, iron was reported to improve the accumulation of Si in the shoots of rapeseed (Diana et al., 2021). Therefore, the increase of Si accumulation in rapeseed plants may be available through the supplement of a high concentration of Si together with iron. Most importantly, the application of Si in the present study exhibited no negative affection on the main agronomic traits and seed quality of rapeseed. This is in accordance with studies in which Si was found to protect plants against many biotic and abiotic stresses without the occurrence of resistance trade-offs and/or growth and yield penalties (Fauteux et al., 2005; Ma and Yamaji, 2006; Laîné et al., 2019). Therefore, Si application may provide a new protective strategy to control SSR in rapeseed. Considering that the highest control efficiency in our field assay was achieved using a Si basal fertilizer plus the spray of a liquid Si fertilizer (RCE = 49.3%), further studies are needed to identify the optimal doses of both the basal fertilizer and the liquid fertilizer to achieve the best control effect on the SSR of rapeseed.

## Data Availability Statement

The datasets presented in this study can be found in online repositories. The names of the repository/repositories and accession number(s) can be found below: BioProject ID PRJNA749126.

## Author Contributions

JM and WQ designed and directed the experiments. YF, YH, XZ, PF, and JW performed most of the experiments and analyzed the data. JW, JL, and WQ discussed the results and improved the manuscript. JM and YF wrote the manuscript. All authors contributed to the article and approved the submitted version.

## Funding

This study was financially supported by the National Nature Science Foundation of China (31971978), the National Key R&D Program of China (2018YFD0100500), the Natural Science Foundation of Chongqing (cstc2019jcyj-zdxmX0012), the Fundamental Research Funds for the Central Universities (XDJK2018AA004), and the Training Program of Innovation and Entrepreneurship for Undergraduate (S202110635220).

## Conflict of Interest

The authors declare that the research was conducted in the absence of any commercial or financial relationships that could be construed as a potential conflict of interest.

## Publisher's Note

All claims expressed in this article are solely those of the authors and do not necessarily represent those of their affiliated organizations, or those of the publisher, the editors and the reviewers. Any product that may be evaluated in this article, or claim that may be made by its manufacturer, is not guaranteed or endorsed by the publisher.

## Abbreviations

ABTS: 2,2-azino-bis-3-ethylbenzothiazoline-6-sulfonic acid
ANOVA: analyses of variance
dai: days after inoculation
DEG: differentially expressed gene
*FDR*: false discovery rate
FW: fresh weight
GO: Gene Ontology
hai: hours after inoculation
KEGG: Kyoto Encyclopedia of Genes and Genomes
PDA: potato dextrose agar
RPKM: reads per kilobyte per million mapped reads
SSR: sclerotinia stem rot.
