# Supplementary material for: Silicon Alleviates the Disease Severity of Sclerotinia Stem Rot in Rapeseed
Source: Front Plant Sci. 2021 Sep 13;12:721436. doi: 10.3389/fpls.2021.721436 (PMC8475755; doi:10.3389/fpls.2021.721436)
Supplement: Supplementary file 1 [file Table_1.DOCX]

**Supplementary Table S1** Primer sequences for qRT-PCR

| **Gene ID** | **Pathway** | **Annotation** | **Primer sequences for qRT-PCR** | |
| --- | --- | --- | --- | --- |
|  |  |  | **Forward (5’→3’)** | **Reverse (5’→3’)** |
| gene56913 | Phenylpropanoid biosynthesis | Peroxidase 58 | GATTTGGTCGCTTTATCTGGTG | AAGATTTACTAAGGCGGTGGG |
| gene116475 | Phenylpropanoid biosynthesis | Cinnamyl alcohol dehydrogenase 8 | TGGGAAGCAAAGTGACTAGATT | CTTGAATCCGTACGTTTGGATC |
| gene116444 | Phenylpropanoid biosynthesis | Phenylalanine ammonia-lyase 2 | ATTGACGTTTCGAGGAACAAAG | GCTAAACGTGTGTTGTCCATAG |
| gene89271 | Phenylpropanoid biosynthesis | Cinnamoyl-CoA reductase 2 | AGTATCCGCTTCCTATCAAGTG | GCTCTTGACAGATTCGTAGAGA |
| gene5158 | Glutathione metabolism | Glutathione S-transferase U8 | AAATCGATAGCTGAGTCTCTGG | CAAGCCTTCCACAATGTTAACA |
| gene80471 | Glutathione metabolism | Glutathione S-transferase U17 | GAGTTCTTCTTCTCGGTTCTCA | GATTTGAGGTTGAGAGCGATTC |
| gene93025 | Glutathione metabolism | Glutathione synthetase | TCTTCCAAAAAGCTTACGGTTG | TCGTAGACCAGTTTCTGAACAA |
| gene28331 | Pathogenesis-related gene | Pathogenesis-related protein 5 | TCATAGCTACAGGCACAGTTAC | GTTGAGTTGTACACCGTTGTC |
| gene98808 | Pathogenesis-related gene | Endochitinase EP3 | TCGGTTGATGAAATTGTCACAC | CGGCTGTAAAATCCATTACCAG |
| gene1163 | Pathogenesis-related gene | Endochitinase CHI | ATGTTCGCTCGAGCTTTAAATC | TTGGCAGCTTTCTCTGTAGAAT |
| gene13646 | Ubiduinone and other terpenoid-quinone biosynthesis | Probable aminotransferase TAT3 | GGAAACACTGCAACTAGTGATG | TCCGTTTAGATAATCCGCTACC |
| gene88819 | Ubiduinone and other terpenoid-quinone biosynthesis | Isochorismate synthase 1 | ATGAGAACAGTAACAGAGGCTC | CAGCGATTTTACCGTTAGGATC |
| *BnActin7* |  | *Brassica napus* actin-7 | CCCTGGAATTGCTGACCGTA | TGGAAAGTGCTGAGGGATGC |
