# Supplementary material for: Silicon Alleviates the Disease Severity of Sclerotinia Stem Rot in Rapeseed
Source: Front Plant Sci. 2021 Sep 13;12:721436. doi: 10.3389/fpls.2021.721436 (PMC8475755; doi:10.3389/fpls.2021.721436)
Supplement: Supplementary file 2 [file Table_2.DOCX]

**Supplementary Table S2** Sequencing data statistics

| **Sample** | **Description** | **Clean reads** | **Clean bases** | **GC Content** | **%≥Q30** |
| --- | --- | --- | --- | --- | --- |
| L-S | Rapeseed leaf absorbed K_2_SO_4_ for 24 hours | 20,899,817 | 6,260,121,282 | 47.45% | 92.79% |
| L-Si | Rapeseed leaf absorbed K_2_SiO_3_ for 24 hours | 25,687,376 | 7,688,173,592 | 47.55% | 93.63% |
| L-S-s | Rapeseed leaf at 24 hai by ‘1980’ on L-S | 21,250,041 | 6,350,907,454 | 47.12% | 93.05% |
| L-Si-s | Rapeseed leaf at 24 hai by ‘1980’ on L-Si | 20,325,855 | 6,080,289,798 | 46.96% | 92.92% |
