# Supplementary material for: Silicon Alleviates the Disease Severity of Sclerotinia Stem Rot in Rapeseed
Source: Front Plant Sci. 2021 Sep 13;12:721436. doi: 10.3389/fpls.2021.721436 (PMC8475755; doi:10.3389/fpls.2021.721436)

**Supplementary Figure S1** Resistance performance of rapeseed plants under different treatments in the field experiment. Arrows indicate infected plants


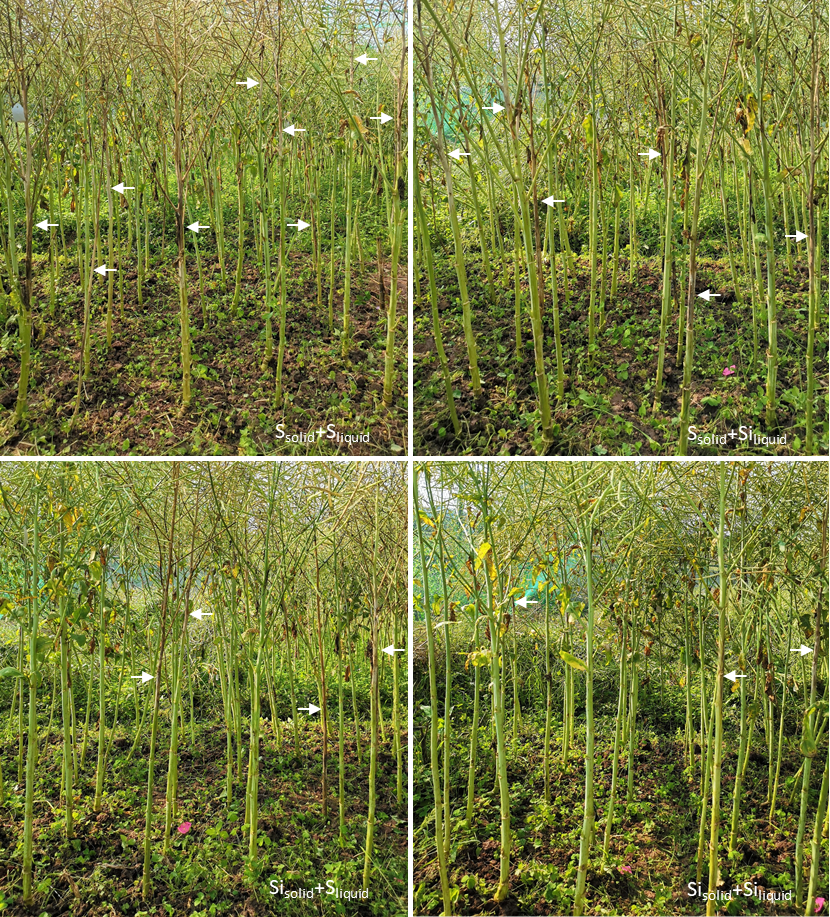

Supplement: Supplementary file 3 [file Data_Sheet_1.DOCX]
