# Supplementary material for: Silicon Alleviates the Disease Severity of Sclerotinia Stem Rot in Rapeseed
Source: Front Plant Sci. 2021 Sep 13;12:721436. doi: 10.3389/fpls.2021.721436 (PMC8475755; doi:10.3389/fpls.2021.721436)
Supplement: Supplementary file 4 [file Data_Sheet_2.DOCX]

**Supplementary Figure S2** Expression patterns of 12 DEGs involved in defense associated pathways. The relative expression of genes in qRT-PCR was indicated by bar with ordinate on the left; the FPKM in RNA-seq. were presented by hollow circles with ordinate on the right side. P: phenylpropanoid biosynthesis; G: glutathione metabolism; U: ubiquinone and other terpenoid-quinone biosynthesis; PR: pathogenesis-related genes.
